# Should we reconsider the apoptosis as a strategic player in tissue regeneration?

**DOI:** 10.7150/ijbs.36362

**Published:** 2019-07-25

**Authors:** Bruna Codispoti, Irina Makeeva, Jamal Sied, Caterina Benincasa, Salvatore Scacco, Marco Tatullo

**Affiliations:** 1 Marrelli Health, Tecnologica Research Institute, Biomedical Section, Street E. Fermi, Crotone, Italy; 2 Department of Therapeutic Dentistry, IM Sechenov First Moscow State Medical University, Moscow, Russia; 3 Advanced Technology Dental Research Laboratory, Faculty of Dentistry, King Abdul Aziz University, KSA and Director of CODE-M, Center of Dental Education and Medicine, Pakistan; 4 Dept. of Basic Medical Sciences, Neurosciences and Sense Organs, University of Bari, Italy

**Keywords:** Apoptosis, Regenerative Medicine, Mesenchymal Stem Cells, Cell Death

## Abstract

Apoptosis plays a central role in organs development and homeostasis. Impaired regulation of this process is often associated with the onset of several human diseases, such as developmental disorders and cancer. The last scientific investigations have discovered interesting connections between apoptosis, stem cells, tissue regeneration and cancer. The role of "programmed cell death" in stem cells and tissue engineering is extremely promising; in fact, it holds great potential for regenerative purposes. However, several questions still remain unsolved: do we really know all the main molecular actors able to switch ON/OFF the apoptosis? Is it possible to modulate these players, to obtain a predictable regeneration of tissues and organs? But primarily: should we reconsider the apoptosis as a strategic player in tissue regeneration? In this topical review, we have carefully examined the most recent discoveries about the role of apoptosis in stem cells and, specifically, in mesenchymal stem cells. The pivotal molecules involved in the activation and inhibition of the apoptotic pathways will be carefully described, with the aim to shed an overall light on the complex scenario of stem cell life and death, and on a novel strategy for tissue regeneration.

## Introduction

Apoptosis plays a central role in organs development and homeostasis. Impaired regulation of this process is often associated with the onset of several human diseases, such as developmental disorders and cancer 1-3. In recent years, the scientific community has investigated on apoptosis, because of interesting connections between apoptosis, stem cells, tissue regeneration and cancer. It is clear that apoptosis plays a central role in organs development and homeostasis: in this light, the molecules and the cell organelles involved in the activation and inhibition of the apoptotic pathways have been carefully described in the scientific literature, however, some aspects still remain unclear 4. In this scenario, mitochondria have been demonstrated to have a central role in the apoptotic pathways. Bcl-2 proteins regulate the release of specific molecules in the mitochondrial intermembrane space; subsequently, these proteins migrate to the cytosol, where they activate the caspase cascade. The involvement of mitochondria is essential in the "intrinsic apoptotic pathway", also called “mitochondrial pathway”; in fact, in the mitochondria, the anti-apoptotic and pro-apoptotic processes interact and trigger the release of molecular signals able to activate the cytosolic caspases 5. These organelles can move the cytochrome-c into the cytosol, thus promoting the assembling of the apoptosome; this multiprotein complex stimulates the activation of caspase-9 from procaspase-9, that is able to cleave the caspase-3, thus resulting in cell apoptosis 6. In the extrinsic pathway, the starting signal is obtained through the close interaction among the ligands with the "death receptors", a group of proteins belonging to the "tumor necrosis factor" (TNF) receptor family. Upon such binding, the death-inducing signaling complex (DISC) is created, and it can induce the activation of caspase-8, that consequently cleave also the caspase-3, thus leading to the apoptosis 7.

Apoptotic cells undergo segmentation of the nucleus and alteration of their membrane, with a consequent formation of the so-called "apoptotic bodies", typically phagocytized by immune or surrounding cells 8.

The scientific discussion on the role of apoptosis in tissue regeneration can be considered as a relatively novel topic; on the other hand, the role of mesenchymal stem cells (MSCs) in regenerative procedures have been widely demonstrated. MSCs have been isolated from many human tissues, and they promise useful applications in different pathological conditions. MSCs have the ability to differentiate into the three classical mesenchymal lineages: adipogenic, osteogenic and chondrogenic 9; however, several researches reported the interesting ability of such cells to be also differentiated toward several other cell types, including neurogenic and cardiogenic phenotypes.

MSCs express the surface markers CD73, CD90, and CD105, but they lack the expression of CD34, CD45, and HLA-DR hematopoietic antigens. After their first discovering in human bone marrow, MSCs have been isolated from several other tissues, including adipose tissue, fetal structures as umbilical cord and, placenta, and the oral cavity 10,11.

The potential of MSCs to differentiate into osteoblasts have been exploited for bone regeneration 12. There are enough pieces of evidence that highlight how MSCs can differentiate into a large variety of other mature cells 13: this fact stimulated the worldwide researchers to speculate a promising role for MSCs in human regenerative therapy. Furthermore, these cells play a strategic role in the local immunomodulation, useful for counteracting adverse immunologic reactions after transplantation of tissues and organs 14.

In the last years, MSCs have been used in clinical trials targeting the regeneration of damaged tissues, as well as in the treatment of several pathologies, such as multiple sclerosis (MS), Crohn's disease, diabetes and graft-versus-host disease (GVHD). Numerous clinical trials are ongoing, to better understand the different aspects related to efficacy and safety of MSCs for clinical use 15.

In this topical review, we have carefully examined the most recent discoveries about the role of apoptosis in stem cells and, specifically, in mesenchymal stem cells. The pivotal molecules involved in the activation and inhibition of the apoptotic pathways will be carefully described, to shed an overall light on the complex scenario of stem cell life and death, and on a novel strategy for tissue regeneration.

A literature searching strategy has been used to select the papers to be included in this review. The most used searching engines, such as Medline and Google Scholar has been used by searching for the following keywords: "Apoptosis AND Regeneration", "Apoptosis AND Stem Cells", "Apoptosis AND MSCs", "Apoptosis AND Disease" and "Apoptosis AND Tissue repair". The authors have carefully revised the title, the abstract, and the main text for each of the selected papers. Finally, the papers specifically correlated to our aim were included in this review.

## Apoptosis in pathology and tissue repair

Based on the central role of apoptosis in maintaining the body homeostasis, it is not surprising that specific alterations of this process may lead to several pathological conditions. Numerous diseases are related to a massive cell death, which determines severe damages with severe consequences for patients' health. In liver failure induced by drugs or alcohol, viral infections, stroke, heart infarction, and cachexia 16-19, a general cell loss typically occurs and certainly may contribute to the increase of patient mortality 20.

In cancer pathogenesis, several alterations of different proteins involved in apoptotic pathways, including the Bcl-2 Family proteins, the death receptor, the apoptosome, and the aberrant caspase activity, are commonly found in neoplastic cells. The mutation of p53 gene, which is fundamental in activating programmed cell death in response to different stimuli, represents the major alteration found in the human tumors: this is indicative about the importance of apoptosis in neoplastic transformation.

Degenerative disease of the brain, including Alzheimer's disease 21, Parkinson's disease 22 and amyotrophic lateral sclerosis 23 could exert an involvement of caspase proteins in their pathogenesis. Moreover, autoimmune diseases, such as systemic lupus erythematosus and the autoimmune lymphoproliferative syndrome, may depend on alteration of immune homeostasis and maintenance of immune tolerance that are strongly related to apoptosis 24.

Interesting studies have pointed out the central role of apoptosis in tissue repair 25. Wound healing is a multistep process, consisting of different phases: in a first step, the open wound triggers an intense inflammatory response, able to protect the biological site against the external microorganisms; furthermore, several inflammatory cells, such as neutrophils and macrophage, typically produce cytokines, chemokines and growth factors that stimulate the migration and the proliferation of fibroblast cells 25. When the inflammatory response declines, this new phase is characterized by the proliferation of collagen-producing cells, and by the contemporary disappearing of inflammatory cells after massive apoptosis 26.

Experimental studies reported that apoptosis occurring within the inflammatory phase of wound healing could be mediated by specific cytokines, such as tumor necrosis factor-α and interleukin-1, released from immune cells: these mediators promote a local response that activates the programmed cell death 27. Alternatively, the apoptosis of inflammatory cells could be p53-mediated 28. During the proliferative phase of tissue repair, fibroblasts exert their function of collagen deposition, on the other hand, in the last phase of wound maturation, fibroblasts, and residual endothelial cells undergo apoptosis 29.

Some *in vitro* researches propose that apoptosis of fibroblasts and vascular cells could be induced by c-myc, that promotes the interaction of fas and Fas-ligand on cell membranes 30; on the other side, the interferons could mediate apoptotic pathway activation 31.

Numerous animal models have clarified the complex apoptotic mechanisms, including the wide range of molecular elements involved and the multitude of pathways implicated. Apoptosis was first characterized in *C. Elegans;* in a second stage, different biological systems, including Drosophila and mammals, allowed further investigations 32. Currently, mathematical models and single-cell analysis represent innovative tools for a better understanding of the complex mechanisms of cell death 33.

## Apoptosis and regeneration

The programmed cell death has a remarkable significance in regulating the correct cell replication in damaged tissue. Worldwide labs have been increasingly involved in research programs aimed to clarify the intriguing role of apoptosis in stem cell function during tissue regeneration **(Table 1).**

The importance of the apoptotic pathways is particularly interesting in the gastrointestinal tract: in fact, a limited number of stem cells cyclically undergo apoptosis because of a mechanism controlled by a p53-independent way. On the other hand, in response to genotoxic stimuli, stem cells residing in the gastrointestinal duct undergo p53-dependent apoptosis. This different regulation pathway could partially explain the lower prevalence of cancer in such tissues with high-rate cell turn-over 1. In the intestinal epithelium, the high-rate cell division is controlled by the Wnt-signalling that typically promotes the self-renewal ability of MSCs; however, the MSC self-renewal should be balanced with an analog rate of apoptosis, and the molecular mechanisms deputed to regulate such process involve the pro-apoptotic protein ARTS. ARTS protein is an atypical mitochondrial protein able to modulate TGF-beta-induced apoptosis and to induce cell killing by several pro-apoptotic factors: when ARTS protein is deactivated, this can preserve the intestinal cells from apoptosis, and, on the other hand, this can promote the in-vitro formation of complex organoids through the activation of specific Wnt-signalling.

During the MSC apoptosis, ARTS protein is activated, and it passes from the mitochondria to the cytoplasm: there, ARTS blocks XIAP, a potent apoptosis inhibitor. In recent studies, mice models deficient for ARTS expression showed significant resistance against intestinal tissue damage, and those animals showed improved tissue regeneration, mainly mediated by the blocking of XIAP 34. Fuchs et al. showed that mice lacking the Sept4/ARTS gene showed higher levels of hair follicle stem cells. Surprisingly, mice lacking the Sept4/ARTS gene exhibited also a significant enhancement in wound healing ability and in hair follicle regeneration. The inhibition of the target-protein for ARTS reverted these phenomena and reduced the previously observed wound healing. The main results reported in several international studies on ARTS-mediated apoptosis have clearly indicated that the previously described pathways could represent a promising tool, able to improve tissue repairing and wound healing 35.

Apoptosis occurring during the tissue development involves at the same time entire clusters of cells, in order to ensure proper growth of immature tissues; in this context, we can report as an example what commonly happens during the catagenic-phase of the hair follicles. The "cluster apoptosis" is regulated by a feedback mechanism, called "apoptosis-induced-apoptosis" (AIA) 36. During this biological process, the first cells undergo apoptosis release specific molecular signals, such as those belonging to TNF family, to the surrounding cells: these cells are thus induced to follow the same fate, undergoing apoptosis too. The starting apoptosis signaling is supposed to be amplified through the activation of the c-Jun N-terminal kinase (JNK) pathway 37. In the first studies, AIA was extensively studied in Drosophila model, and the following investigations further described this biological phenomenon also in mammals. In humans, AIA takes place during the development of the tissues and under specific pathological conditions, such as the wide cell death associated with strokes 38.

The tissue regeneration that occurs after any traumatic wound, typically consists of a proliferative reply by local cells, in order to replace the damaged tissue; this physiologic mechanism is called "compensatory proliferation". Apoptotic cells produce mitogenic signals able to trigger this compensatory proliferation: this pivotal biological mechanism is called "apoptosis-induced compensatory proliferation" (AIP). Apoptosis-induced proliferation is an advantageous process for the organism, as it permits the elimination of damaged cells that could develop genetic mutations or cancer. Furthermore, AIP allows replacing those atypical cells with a new progeny of healthy cells. Alterations of this compensatory process may potentially result as pathological for the body 39.

AIP has been first discovered in the study-model Drosophila melanogaster; several studies have confirmed that apoptotic cells can secrete different mitogenic signals, able to trigger the cell mitosis of neighboring cells 40.

Pro-apoptotic signals can also induce the production of reactive oxygen species (ROS), that have been demonstrated to be essentials for an effective wound healing and the local recruitment of immune cells at the site of injury 41.

In Xenopus tadpole, tail amputation induces a profuse production of ROS; these amputation-induced ROS can activate the Wnt/β-catenin and FGF signaling, and this starts an active tissue regeneration 42.

Hydra is also able to regenerate any part of its body. Following a surgically created trauma, this polyp rapidly regenerates its head, and it undoubtedly seems to be promoted by massive apoptosis. In some tissues of hydra, on the other hand, the apoptosis is very limited: consequently, the tissue regeneration is markedly slower. Forced induction of apoptosis also in the foot-related tissues fatally promotes the rapid formation of a second head. Conversely, the inhibition of the apoptosis blocks any regrowth of amputated organs. These impressive examples about the apoptosis in different animal models are strongly suggestive about the strategic role of the releasing of WNT3 signal by apoptotic cells: this is the main engine that induces tissue regeneration 43.

Oxidative stress is a well-known trigger for different pathological conditions 44,45, ROS have also a central role in the apoptosis induction. The pathways involved in the apoptotic mechanism of MSCs have been widely investigated during experimental assays with H2O2: hydrogen peroxide is a quite common exogenous oxidative stress model for MSCs, because of the good chemical properties showed by H2O2, such as the good solubility in aqueous and non-aqueous solutions and its extensive half-life 46.

The administration of H2O2 promotes apoptosis in MSCs, in a time- and dose-dependent manner. This treatment is able to induce the activation of p38 and JNK signaling, however, it does not affect the ERK1/2 - MAPK pathway and the death receptor signaling. To better describe these molecules: the P38 can start the mitochondrial cell death pathway and the ER-initiated (endoplasmic reticulum-initiated) cell death pathways, that lead to early apoptosis; the JNK activation, instead, is involved in the late apoptosis 47.

## Apoptosis and stem cells

Current studies focused on tissue regeneration are based on the application of alternative techniques: some interesting ones are the using of Platelet-Rich Fibrin (PRF) for the management of uncommon inflammatory diseases 48, and the application of useful prosthetic devices for patients' rehabilitation 49, 50. Furthermore, recent efforts allowed to monitoring the healing processes by alternative techniques of analysis 51. On the other hand, several studies also confirmed the enormous potential of stem cell therapy in regenerative medicine.

Apoptosis-related caspases are involved in the differentiation and growing of stem cells.

Deficiency of the effector caspase 3 in mice, increases the proliferation of hematopoietic stem cells and delays their differentiation; furthermore, in primarily derived neural stem cells showing a lacking of caspase 3, it reduces the physiological differentiation into neurons 52.

In embryonic stem cells, the overexpression of caspase 3 induces their differentiation by down-regulation of Nanog signaling; Nanog is a well-known target of caspase 3, and the expression of a cleavage-resistant form of Nanog has been demonstrated in self-renewal increasing of embryonic stem cells 53. Caspase-3-deficient mice showed a retarded ossification and a reduced bone mineral density. Bone marrow-derived MSCs from mutant mice displayed a deficit in osteogenic differentiation that could be partially explained by the detected over-activation of the TGF-beta/Smad2 pathway 54.

Caspase 3 is also involved in cell proliferation and the regulation of organs size.

In sebaceous glands, the proliferating cells may express activated caspase 3 without any sign of apoptosis. The caspase 3 induces nuclear translocation of the central regulator protein of organ size: the Yes-associated protein; thus, caspase 3 inhibition reduces cell proliferation and sebaceous gland dimension 55.

Apoptosis is also involved in the MSC-mediated immunomodulation in transplants. In a mouse model of graft-versus-host disease (GVHD), the infusion of apoptotic MSCs activates phagocytic cells that produce the immunosuppressive mediator indoleamine 2,3-dioxygenase, after the incorporation of injected apoptotic cells 56. This *in vivo* evidence, underlines that also the *in vitro* induction of apoptosis, mediated by caspase 3 activation, could represent a valuable tool for the rapid down-regulation of the immune response in GVHD patients. Furthermore, several studies have supported the concept that host cytotoxic cells can induce apoptosis of injected MSCs: this allows that transplanted MSCs does not engraft into host recipient site, although they may continue to act with their immunomodulatory activity.

Apoptotic bodies are atypical extracellular vesicles produced by dying cells (ApoEVs): we previously described the ability of ApoEVs, derived from MSCs, to modulate the immune response in autoimmune conditions, in antitumor immunity and infective diseases 57.

Exogenous transplanted apoptotic bodies have been demonstrated to promote the MSCs proliferation and the osteogenic and adipogenic differentiation. The basal mechanism reported by the researchers was related to the activity of exogenous apoptotic bodies, strategically able to reuse the molecular inhibitor of Axin, finally resulting in the activation of the WNT-pathway and the self-renewal initiation 58.

In conclusion, the apoptosis of MSCs could be generally started by a severe alteration of the stem cell niche and after local damage, and the apoptosis can be enhanced by the activation of caspase 3-related pathway 59.

## Conclusions and future insights

Nowadays, cell therapy often requires *in vitro* cell manipulation and cell expansion 60 to obtain an adequate number of cells to ensure successful transplantation in the recipient site. On the other hand, any stem cell transplantation should be carefully monitored because of their moderate risk to observe aberrations and preneoplastic transformations. Injuries and cancers, in fact, share common molecular mechanisms; fatally, stem cells are involved in both tissues repairing and tumors pathogenesis phenomena. Anti-apoptotic proteins are, on the other side, deeply correlated with stem cells survival 61.

Lastly, the discovery that dying cells exert an incredible regenerative potential has increasingly stimulated scientific research to better understand the fascinating cellular and molecular mechanisms of programmed cell death and of tissue regeneration 62.

Although AIP could seem to represent a valuable tool for regenerative medicine, it may be certainly considered as a double-edged sword, because of the potential induction of tumors in in-vivo models. Radiotherapy and chemotherapy are able to eradicate tumor-related cells by triggering their death; however, such dying cells might undergo AIP, and thus they can produce mitogenic stimuli that might contribute to metastasis and relapses of primary tumors 63.

On the other hand, the complex mechanism of AIA could be, at least partially, responsible for the bystander effect, where non-irradiated cells dye, just like the tumors cells 64.

**Figure 1:**
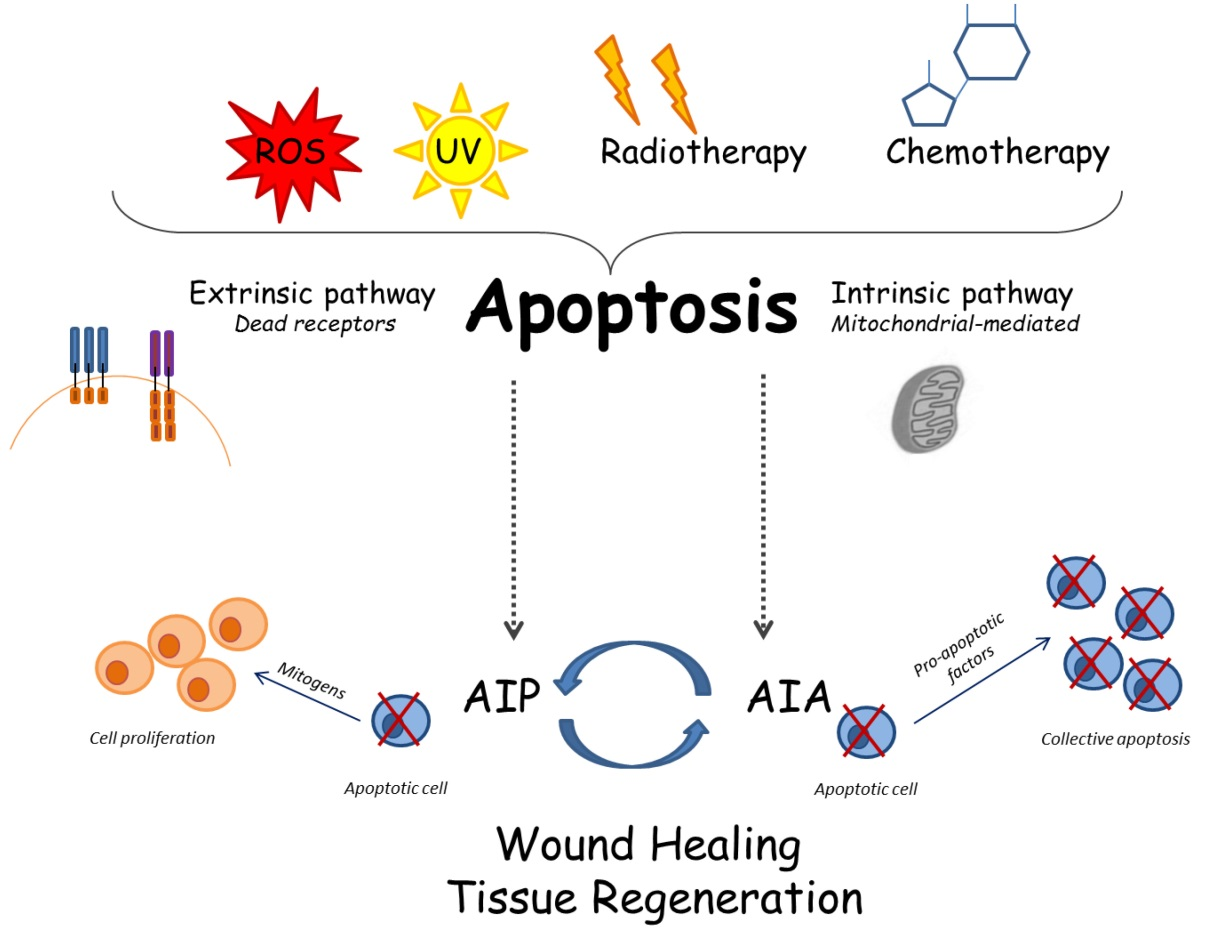
** Cellular and molecular apoptotic mechanisms:** several stimuli can trigger apoptosis; consequently, the apoptosis may follow the intrinsic or the extrinsic way. The recently described mechanisms called AIP and AIA may be involved in complex interactions among apoptosis and tissue regeneration.

Some interesting studies have recently linked the two mechanisms AIA and AIP; in fact, they seem to be opposed pathways, but they indeed work in close correlation, thus demonstrating that apoptosis is a mechanism more complex than we are commonly used to know. It would seem that AIA works to increase the apoptotic cells and that these cells consequently produce mitogenic signals able to induce stronger cell proliferation 65 **(Figure 1).**

In this review, we tried to shed light on the key molecular mechanisms that promote and/or block apoptotic pathways, describing the studies carried out on animal models that have illustrated the correlation among apoptosis and WNT, JNK p53, p38 pathways, often mediated by the recently discovered ARTS and XIAP proteins; these peptides are able to switch ON/OFF apoptosis, in order to regulate the tissue development and regeneration. The provided experimental evidence could be potentially exploited in a clinical scenario, in order to suggest a predictable regeneration of tissues and organs by manipulating of the apoptotic process.

The everchanging knowledge of cell biology still remains largely obscure; however, we can take into account that an indissoluble link between cell apoptosis and tissue regeneration do exist, and it has been consistently confirmed in the last years. This extremely interesting correlation between apoptosis and regeneration seems to suggest different landscapes from the old theory about the role of apoptosis as a process only aimed at cell death, thus considering this biological process as a pivotal player for tissue regeneration and organs homeostasis.

## Figures and Tables

**Table 1:** Experimental evidences of the main apoptotic events

Apoptotic events	Experimental systems	Molecular mechanisms	Induced effects	References
p53-dependent apoptosis	*In vivo*: cells of the gastrontestinal tract	p-53 protein activation	Control of stem cell proliferation	Potten. *Oral Dis* (2001)
Apoptosis	*In vitro*: *Sept4*/ARTS^-/-^ crypt cells	Activation of ARTS by induction of Wnt signalling and block of XIAP	ART protects intestinal cells from apoptosis, increments cell proliferation and produces organoids, *in vitro*	Koren et al. *Nat Commun* (2018)
	*In vivo*:* Sept4*/ARTS^-/-^ mice	ARTS mediates apoptosis independently of Wnt signaling	ARTS loss protects mice from formation of cysts and inflammatory colitis	
Apoptosis	*In vivo*: *Sept*4/ARTS^-/-^ mice	ARTS inactives XIAP	ARTS has the ability to heal wounds and regenerate the hair follicle by regulation of stem cell regeneration	Fuchs et al. *Science* (2018)
Apoptosis-Induced-Apoptosis (AIA)	*In vivo*:			Perez-Garijo et al. *Elife* (2013)
	*-Drosophila*	Activation of the JNK pathway	Cell death in a non-autonomous way	
	*-Mice*	TNF molecular signal	The cells that first take the path of apoptosis induce the surrounding cells to apoptosis	
Apoptosis-induced compensatory proliferation (AIP)	*In vivo*: *Drosophila melanogaster*	Activaction of Orthologue protein of WNT BMPs and EGF	Apoptotic cells produce mitogenic signals that trigger compensatory proliferation	Fan. et al. *PLoS Genet* (2014)
Apoptosis-induced compensatory proliferation (AIP)	*In vivo*: *Drosophila melanogaster*	Activation of the Jun N-terminal kinase and p53 mediated signal through the initial caspase Dronc. ROS formation	Decapentaplegic and Wingless mitogens are induced and trigger compensatory proliferation in surrounding cells and the recruitment of immune cells on the site of injury	Fan et al. *Trends Cell Biol* (2008)
Apoptosis-induced compensatory proliferation (AIP)	*In vivo*: *Xenopus tadpole*	ROS activate Wnt/β-catenin and FGF signalling	The signal produces the activation of numerous pathways, including the Wnt, Fgf, BMP, notch, and TGFβ pathways, that induce cell proliferation	Love et al. *Nat Cell Biol* (2013)
Apoptosis-induced compensatory proliferation (AIP)	*In vivo*: *Hydra*	ROS activate Wnt3	WNT activates β-catenin and induces proliferation of surrounding cells	Chera et al. *Dev Cell (*2009)
Apoptosis and regeneration	*In vitro*: MSCs	Activation of p38 and JNK signalling	P38 initiates cell-death pathways that lead to early apoptosis; JNK activation, instead, is involved in the late apoptosis	Wei et al. *J Cell Biochem* (2010)
Apoptosis and regeneration	*In vitro*: MSCs	Caspase 3 activation	Caspase-3 alters signal transduction by limiting activation of the Ras-Raf-MEK-ERK pathway, the lack of caspase 3 reduces the differentiation in neurons	Janzen et al. *Cell Stem Cell* (2005)
	*In vivo*: Mice	Caspase 3 deletion	The lack of the caspase-3 effector in mice increases the proliferation of hematopoietic stem cells and slows their maturation	
Apoptosis and regeneration	*In vitro*: Embryonic stem cells	Espression of Caspase 3	Induce differentiation by down-regulation of Nanog signalling	Fujita et al. *Cell Stem Cell (*2008)
Apoptosis and regeneration	*In vivo*: (Casp3(-/-) and Casp3(+/-)) mice	The overactivated TGF-beta/Smad2 signalling pathway and the upregulated expressions of p53 and p21	The shortage of Caspase 3 retards ossification and reduced bone mineral density	Miura et al. *J Clin Invest* (2004)
Apoptosis and regeneration	*In vitro*: Sebaceous gland cells	Caspase-3 induces the translocation of YAP protein	Caspase-3 activation leads to an increase in the size of the YAP-dependent organ; Caspase-3 inhibition reduces cell proliferation and sebaceous gland dimension	Yosefzon et al. *Mol Cell* (2018)
Apoptosis	*In vivo*: GVHD mouse model	Infusion of apoptotic MSCs	Immunomodulation by indoleamine 2,3-dioxygenase released by phagocytes	Galleu et al. *Sci Tran Med* (2017)
Apoptosis	*In vitro*: Apoptotic bodies (ApoEVs)	Infusion of ApoEVs	Regulation of immune response	Codispoti et al. *J Clin Med* (2018)
Apoptosis	*In vivo*: Fas-deficient MRL/*lpr* and *Caspase 3*^-/-^ mice	Activation of Wnt pathway	Engulfment of exogenous apoptotic bodies by recipient MSCs, that reuse molecular inhibitor of Axin finally resulting in activation of Wnt pathway and self-renewal initiation	Liu et al. *Cell Res* (2018)
Apoptosis	*In vitro*: human bone marrow-derived MSCs	Non-adherent culture conditions	Removal from adherent culture contributes to apoptosis in human bone marrow mesenchymal stem cells	Deng et al. *Mol Med Rep* (2017)
Apoptosis	*In vivo*: Zebrafish	Activation of TNF- pathway	TNFα/TNFR1 signalling pathway is required for the fin regeneration	Nguyen-Chi et al. *Cell Death Dis* (2017)
